# A novel dimension reduction algorithm based on weighted kernel principal analysis for gene expression data

**DOI:** 10.1371/journal.pone.0258326

**Published:** 2021-10-13

**Authors:** Wen Bo Liu, Sheng Nan Liang, Xi Wen Qin

**Affiliations:** 1 School of Mathematics and Statistics, Qiannan Normal University for Nationalities, Duyun, Guizhou, China; 2 Key Laboratory of Complex Systems and Intelligent Computing, Qiannan Normal College of Nationalities, Duyun, Guizhou, China; 3 School of Mathematics and Statistics, Changchun University of Technology, Changchun, Jilin, China; Torrens University Australia, AUSTRALIA

## Abstract

Gene expression data has the characteristics of high dimensionality and a small sample size and contains a large number of redundant genes unrelated to a disease. The direct application of machine learning to classify this type of data will not only incur a great time cost but will also sometimes fail to improved classification performance. To counter this problem, this paper proposes a dimension-reduction algorithm based on weighted kernel principal component analysis (WKPCA), constructs kernel function weights according to kernel matrix eigenvalues, and combines multiple kernel functions to reduce the feature dimensions. To further improve the dimensional reduction efficiency of WKPCA, t-class kernel functions are constructed, and corresponding theoretical proofs are given. Moreover, the cumulative optimal performance rate is constructed to measure the overall performance of WKPCA combined with machine learning algorithms. Naive Bayes, K-nearest neighbour, random forest, iterative random forest and support vector machine approaches are used in classifiers to analyse 6 real gene expression dataset. Compared with the all-variable model, linear principal component dimension reduction and single kernel function dimension reduction, the results show that the classification performance of the 5 machine learning methods mentioned above can be improved effectively by WKPCA dimension reduction.

## 1 Introduction

DNA is organized structurally into chromosomes and functionally into genes, which are essentially pieces of DNA containing genetic information [1]. In humans, genes carry genetic information to express hair and eye colour, among many other traits, as well as information about when the body’s cells grow, divide and die. When a gene is turned on, this is called gene expression. Genetic mutations in normal cells of the human body are closely related to environmental stimuli, age, smoking, diet and other external factors, which can lead to the uncontrolled reproduction of normal cells and, ultimately, to cancer (malignant tumours) [2]. In February 2018, the National Cancer Center of China released the registration data of the National Cancer Registry for 2014, which indicated that there were approximately 3.804 million cases of cancer in 2014, including approximately 2.114 million men and 1.69 million women [3]. The development of sequencing technology has had a huge impact on cancer research, enabling researchers to analyse the expression levels of thousands of genes in a collaborative manner and to correlate gene expression patterns with clinical phenotypes, resulting in multiple tumour gene expression profiles. How to effectively analyse tumour gene expression data and how to mine and discover the information and knowledge contained therein is a hot topic in bioinformatics research. This could help distinguish cancer from normal tissue, predict cancer outcomes, detect cancer recurrence and monitor cancer treatment responses. However, gene expression data have the characteristics of high dimensionality and small sample sizes. Each sample records the expression levels of all the detectable genes in the histocyte, but only a few genes are actually related to the sample categories. These genes contain classification information about samples, which are known as "classification feature genes". At present, most research concerns how to select these informative genes from thousands of genes, which is the problem of feature selection. Many researchers have done a great amount of fruitful work in unsupervised [4–6] semi-supervised [7–9] and supervised [10–12] gene feature selection. Different from feature selection, this paper mainly studies the dimension reduction of gene expression data from the perspective of feature extraction to improve the recognition rate of sample categories. Feature extraction is based on known features and obtains a subset with lower dimensionality and fully represents the original features through a specific algorithm. Moreover, the features in this subset are independent of each other. The main feature extraction algorithms are as follows.

Principal component analysis (PCA) is one of the most classic feature extraction algorithms. Its basic idea is to use fewer principal components (comprehensive variables) to replace more original features, and these principal components can contain as much information about the original features as possible and are unrelated to each other [13, 14]. PCA is good at processing linear and Gaussian distribution data. To make up for the deficiency of PCA, many related studies have been proposed to improve the PCA algorithm. Compared with PCA, the independent component correlation algorithm (ICA) is more suitable for processing non-Gaussian data. Hyvarinen [15, 16] proposed a FastICA algorithm that can quickly find the optimal iteration. This algorithm is a mature linear blind source separation algorithm at present. The above methods are all linear dimension reduction algorithms. In many practical tasks, data often presents a nonlinear distribution. If linear dimension reduction is still adopted, the original low-dimensional structure will be lost. Therefore, some nonlinear dimension reduction techniques are proposed, among which the most typical representative is the nonlinear feature extraction method based on the kernel technique. Schkopf et al. [17] proposed kernel principal component analysis (KPCA), which maps the linear indivisible data in the low-dimensional space to the high-dimensional space through nonlinear mapping and realizes linear divisibility in the high-dimensional space. Mika et al. [18] proposed kernel linear discriminant analysis (KLDA), which combines the kernel function with LDA to extract the features. Xu et al. [19, 20] proposed the fast KPCA method by introducing the key sample idea in the early 1920s. The above nonlinear dimension reduction methods are all based on the single kernel function. To further improve the dimension reduction and classification performance of the kernel methods, multiple kernel learning algorithms are proposed. Gonen et al. classified and summarized these algorithms and concluded that combining multiple kernel functions was better than using single kernel functions through experimental analysis [21]. Zhang et al. introduced the power kernel function, proposed the combined kernel function principal component analysis method, realized data mapping from the low dimension to the high dimension, and then applied the feature extraction to the nonlinear data [22]. Li Proposed pulmonary nodule recognition based on multiple kernel learning support vector machine particle swarm optimization and obtained a better recognition efficiency [23].

Although the above kernel methods have achieved remarkable practical results in many fields, these methods are all single kernel methods based on a single feature space. Because different kernel functions have different characteristics, so that in different applications, the performance of the kernel function is very different, and there is no perfect theoretical basis for the construction or selection of the kernel function. In addition, when the sample features contain Heterogeneous information, the sample size is large, the multi-dimensional data is Unnormalised data or the data is non-flat in the high-dimensional feature space, it is not reasonable to process all the samples by mapping with a single simple kernel. In view of these problems, there are many researches on kernel combination method, namely multiple learning methods. Multiple models are a kind of based kernel learning model with stronger flexibility. Recent theories and applications have proved that using multiple instead of single kernel can enhance the interpretability of decision functions, taking advantage of the feature mapping ability of each basic kernel, and can obtain better performance than single kernel model or single-kernel machine combination model [24].

In view of the advantages of multiple kernel learning, this paper proposes a novel dimension reduction algorithm based on weighted kernel principal component analysis (WKPCA). Its basic idea is to use the vectorization method to calculate the kernel matrix, construct the kernel function weights according to the eigenvalues of the kernel matrix, combine multiple kernel functions, and give the theoretical proof of the weighted kernel functions. Moreover, the t-class kernel function is constructed as a subpart of the weighted kernel function. Through a large number of comparison experiments on 6 real data sets, the results show that compared with the whole variable model, linear principal component dimension reduction and single kernel function dimension reduction, the WKPCA algorithm proposed in this paper can effectively improve the classification prediction performance of the current mainstream machine learning methods.

## 2 Kernel principal component analysis

Traditional dimension reduction methods assume that the mapping from the high-dimensional feature space to the low-dimensional feature space is linear. However, in many practical tasks, nonlinear mapping may be needed to find the appropriate low-dimensional embedding [25]. To compensate for the lack of linear dimension reduction, the nonlinear dimension reduction method based on the kernel function was proposed, among which applications kernel principal component analysis was the most commonly used method. The basic idea is that the original data set by the nonlinear function maps the data to the appropriate high-dimensional feature space, introducing the kernel function whose form is known, so knowing the concrete expression of the nonlinear mapping is not necessary. Then, the kernel matrix and its eigenvectors are calculated, giving the projection of the data set in the high-dimensional space based on the eigenvectors.

Suppose that the original data is *D* = {*x*_1_, *x*_2_, …, *x*_*m*_}, where *x*_*i*_ = {*x*_*i*1_, *x*_*i*2_, …, *x*_*ip*_}′, *m* is the sample size, *i* is the sample number, and *p* is the data dimension. In the high-dimensional feature space, the mapping of *x*_*i*_ is *z*_*i*_ = *ϕ*(*x*_*i*_), and the data set is *D*′ = {*z*_1_, *z*_2_, …, *z*_*m*_}, whose covariance matrix is

$$\Sigma =\sum_{i=1}^{m}z_{i}z_{i}^{T}$$ (1)

The solving goal of KPCA is

$$\left\{\begin{array}{l} z_{j}=\eta_{j}^{T}\phi (x)\\ \left(\sum_{i=1}^{m}z_{i}z_{i}^{T}\right)\eta_{j}=\lambda_{j}\eta_{j},j=1,2,\ldots ,m \end{array}\right.$$

where *ω*_*j*_ is the eigenvector corresponding to the eigenvalue *λ*_*j*_ of Σ, and *z*_*j*_ is the *j*^*th*^ coordinate component after the projection of sample *x*. The key to solving for *z*_*j*_ is how to calculate *ω*_*j*_ and obtain the expression of *ϕ*(*x*). However, *ϕ*(*x*) is often unknown, but it can be replaced through the kernel function, whose form is known. The calculation process of the kernel principal component is as follows.


$$\left(\sum_{i=1}^{m}z_{i}z_{i}^{T}\right)\eta_{j}=\left(\sum_{i=1}^{m}\phi (x_{i})\phi^{T}(x_{i})\right)\eta_{j}=\lambda_{j}\eta_{j}$$ (2)

$$\eta_{j}=\frac{1}{\lambda_{j}}\left(\sum_{i=1}^{m}z_{i}z_{i}^{T}\right)\eta_{j}=\sum_{i=1}^{m}z_{i}\frac{z_{i}^{T}\eta_{j}}{\lambda_{j}}$$ (3)

Here, $\alpha_{i}^{j}=\frac{z_{i}^{T}\eta_{j}}{\lambda_{j}}$.


$$\eta_{j}=\sum_{i=1}^{m}z_{i}\alpha_{i}^{j}=\sum_{i=1}^{m}\phi (x_{i})\alpha_{i}^{j}$$ (4)

Introducing the kernel function

$$\kappa (x_{i},x_{j})=\phi^{T}(x_{i})\phi (x_{j})$$ (5)

Common kernel functions can be found in the literature [26].

Substitute Eqs (4) and (5) into Eq (2) to get

$$\left(\begin{array}{cccc} \kappa (x_{1},x_{1}) & \kappa (x_{1},x_{2}) & \ldots & \kappa (x_{1},x_{m})\\ \kappa (x_{2},x_{1}) & \kappa (x_{2},x_{2}) & \ldots & \kappa (x_{2},x_{m})\\ \vdots & \vdots & \ldots & \vdots \\ \kappa (x_{m},x_{1}) & \kappa (x_{m},x_{2}) & \ldots & \kappa (x_{m},x_{m}) \end{array}\right)\alpha^{j}=\lambda_{j}\alpha^{j}$$ (6)

$$Κ\alpha^{j}=\lambda_{j}\alpha^{j}$$

where K = (*κ*(*x*_*i*_, *x*_*j*_))_*m*×*m*_ is the kernel matrix of *κ*(*x*_*i*_, *x*_*j*_) and $\alpha^{j}={\left(\alpha_{1}^{j},\alpha_{2}^{j},\ldots ,\alpha_{m}^{j}\right)}^{T}$ is the eigenvector corresponding to the *j*^*th*^ largest eigenvalue *λ*_*j*_ of the kernel matrix K.

After projection, the *j*^*th*^ coordinate component of sample *x* is

$$\begin{array}{ll} z_{j} & =\eta_{j}^{T}\phi (x)\\ & =\sum_{i=1}^{m}\alpha_{i}^{j}\phi^{T}(x_{i})\phi (x)\\ & =\sum_{i=1}^{m}\alpha_{i}^{j}\kappa (x_{i},x) \end{array}$$ (7)

where *α*^*j*^ is the normalized vector. It can be seen from Eq (7) that in order to obtain the projection of new samples, all the original data need to be summed, so the calculation cost is large. However, in the algorithm designed in Section 3.2, vectorization programming specific to the R language can be adopted to improve the calculation efficiency [27].

## 3 Weighted kernel function method

### 3.1 Weighted kernel function

To further improve the low-dimensional embedding ability of a single kernel function for the original data and make the selection of the kernel function more flexible, this paper proposes a weighted kernel function method to reduce the dimensionality of gene expression data with super high dimensionality, and its principle is given in the form of the following theorem.

**Theorem 1** [28] Let *X* be the input space, and *κ*(·,·) is a symmetric function defined based on *X* × *X*. Then, *κ*(·,·) is the kernel function if and only if for any dataset *D* = {*x*_1_, *x*_2_, …, *x*_*m*_}, the "kernel matrix" K is always positive semi-definite.

Theorem 1 shows that as long as the kernel matrix of a symmetric function is semi-positive definite, it can be used as a kernel function.

**Theorem 2** If *κ*_1_(*x*, *y*), *κ*_2_(*x*, *y*),…, *κ*_*n*_(*x*, *y*) is the kernel function, then

$$\begin{array}{ll} \kappa (x,y) & =\omega_{1}\kappa_{1}(x,y)+\omega_{2}\kappa_{2}(x,y)+\ldots +\omega_{n}\kappa_{n}(x,y)\\ & =\sum_{i=1}^{n}\omega_{i}\kappa_{i}(x,y) \end{array}$$ (8)

is a kernel function, where $\sum_{i=1}^{n}\omega_{i}=1,\omega_{i}\geq 0,i=1,2,\ldots ,n$.

**Proof**: Supposing that the original data is *D* = {*x*_1_, *x*_2_, …, *x*_*m*_}, the corresponding data matrix can be expressed as *D* = (*x*_*ij*_)_*m*×*p*_.

The kernel matrix of *κ*_*i*_(*x*, *y*) is K_*i*_ = (*κ*_*i*_(*x*_*i*_, *x*_*j*_))_*m*×*m*_; thus, the kernel matrix of *κ*(*x*, *y*) is

$$Κ=\sum_{i=1}^{n}\omega_{i}{Κ}_{i}.$$ (9)

According to Theorem 1, if *κ*(*x*, *y*) is the kernel function, the kernel matrix K is positive semi-definite.

Let K*x* = *λx*. Then *x* and *λ* are the eigenvectors and eigenvalues of K, respectively, so

$$Κx=\sum_{i=1}^{n}\omega_{i}{Κ}_{i}x=\lambda x.$$ (10)

Eq (10) can be expanded

$$\begin{array}{ll} \sum_{i=1}^{n}\omega_{i}{Κ}_{i}x & =\omega_{1}{Κ}_{1}x+\ldots +\omega_{i}{Κ}_{i}x+\ldots +\omega_{n}{Κ}_{n}x\\ & =\omega_{1}\lambda_{1}x+\ldots +\omega_{i}\lambda_{i}x+\ldots +\omega_{n}\lambda_{n}x\\ & =(\omega_{1}\lambda_{1}+\ldots +\omega_{i}\lambda_{i}+\ldots +\omega_{n}\lambda_{n})x\\ & =\lambda x \end{array},$$

therefore

$$\lambda =\omega_{1}\lambda_{1}+\ldots +\omega_{i}\lambda_{i}+\ldots +\omega_{n}\lambda_{n},$$ (11)

where *λ*_*i*_ is the eigenvalue of the matrix *K*_*i*_.

Since the kernel matrices K_1_, K_2_, …, K_*n*_ are positive semi-definite matrices, their eigenvalues *λ*_1_, *λ*_2_, …, *λ*_*n*_ are non-negative. According to Eq (11), all the eigenvalues of K are non-negative, so the matrix K is a positive semi-definite matrix. Because *κ*(*x*_*i*_, *y*_*j*_) = *κ*(*y*_*j*_, *x*_*i*_),

$$\kappa (x,y)=\sum_{i=1}^{n}\omega_{i}\kappa_{i}(x,y)$$

is the kernel function.□

When the weighted kernel function of Eq (8) is used to reduce the dimensionality of the original data, the problem of weight value will be encountered. The basic criterion of weight construction is the ratio of the eigenvalues of each K_*i*_ in the weighted kernel to the sum of all of them. The detailed construction process is as shown below.

Assume that all the eigenvalues of the kernel matrix K_*i*_ are $\lambda_{1}^{i}\geq \lambda_{2}^{i}\geq \ldots \geq \lambda_{m}^{i}$ in sequence, where *i* = 1, 2, …, *n*, *p* is the dimension of the original data set, and *d* is the dimension taken after the reduction of the kernel function. Generally, *d* < *p* or *d* ≪ *p*. The weight of the kernel function is

$$\omega_{i}=\frac{\sum_{j=1}^{d}\lambda_{j}^{i}}{\sum_{j=1}^{d}\lambda_{j}^{1}+\sum_{j=1}^{d}\lambda_{j}^{2}+\ldots +\sum_{j=1}^{d}\lambda_{j}^{n}}=\frac{\sum_{j=1}^{d}\lambda_{j}^{i}}{\sum_{i=1}^{n}\sum_{j=1}^{d}\lambda_{i}^{j}}.$$ (12)

Through the concept of "weighted kernel function dimension reduction efficiency", the value range of the final number *d* of feature extractions is determined.

**Definition** Suppose that the eigenvalues of the kernel matrix K = (*κ*(*x*_*i*_, *x*_*j*_))_*m*×*m*_ are *λ*_1_ ≥ *λ*_2_ ≥ … ≥ *λ*_*m*_ ≥ 0. Then, we determine that

$$R_{j}=\frac{\lambda_{j}}{\sum_{i=1}^{m}\lambda_{j}}$$ (13)

is the dimension reduction efficiency of the kernel discriminant function $z_{j}=\sum_{i=1}^{m}\alpha_{i}^{j}\kappa (x_{i},x)$.

$$R_{d}=\frac{\sum_{i=1}^{d}\lambda_{j}}{\sum_{i=1}^{m}\lambda_{j}}$$ (14)

is the cumulative dimension reduction efficiency of the first *d*(*d* ≤ *m*) kernel discriminant function *z*_1_, *z*_2_,⋯*z*_*d*_. According to the cumulative contribution rate of principal component analysis [29], the number of features *d* after dimension reduction can make *R*_*d*_ reach 0.8 ~ 0.9.

### 3.2 T-class kernel function

The weighted kernel function is the combination of multiple single kernel functions. The selection of a single kernel function will directly affect the dimensional reduction effect of the weighted kernel function. Therefore, we need to try to construct the new kernel function to improve the ability of weighted kernel functions to reduce the dimensionality of high-dimensional data to improve the classification performance of subsequent machine learning algorithms. According to the following Theorem 3 and probability density function of the t distribution, the t-class kernel function can be constructed.

**Theorem 3** [30] Suppose that *f*: *X* → *R* is a bounded continuous integrable function. Then, *k*(*x* − *x*′) = *f*(*x* − *x*′) is a kernel function if and only if its Fourier transform

$$\tilde{f}(\omega )=\int_{-\infty }^{+\infty }f(x)e^{-i\omega x}dx\geq 0.$$

**Theorem 4** When *n* → +∞, the probability density function of the t distribution

$$f(x)=\frac{\Gamma (\frac{n+1}{2})}{\sqrt{n\pi }\Gamma (\frac{n}{2})}{\left(1+\frac{x^{2}}{n}\right)}^{-\frac{n+1}{2}}$$ (15)

is the kernel function.

**Proof**: First, $f(0)=\frac{\Gamma (\frac{n+1}{2})}{\sqrt{n\pi }\Gamma (\frac{n}{2})}>0$. We just have to prove that the Fourier transform is non-negative, as *n* → +∞.

Because $\underset{n\to +\infty }{\text{lim}}f(x)=\underset{n\to +\infty }{\text{lim}}\frac{\Gamma (\frac{n+1}{2})}{\sqrt{n\pi }\Gamma (\frac{n}{2})}{\left(1+\frac{x^{2}}{n}\right)}^{-\frac{n+1}{2}}=\frac{1}{\sqrt{2\pi }}e^{-\frac{x^{2}}{2}}$, we have

$$\begin{array}{ll} \tilde{f}(\omega ) & =\underset{n\to +\infty }{\text{lim}}\int_{X}f(x)e^{-i\omega x}dx\\ & =\int_{X}\underset{n\to +\infty }{\text{lim}}f(x)e^{-i\omega x}dx\\ & =\int_{-\infty }^{+\infty }\frac{1}{\sqrt{2\pi }}e^{-\frac{x^{2}}{2}}e^{-i\omega x}dx\\ & =\int_{-\infty }^{+\infty }\frac{1}{\sqrt{2\pi }}e^{-\frac{x^{2}}{2}}e^{-i\omega x}dx\\ & =\sum_{n=0}^{\infty }\frac{{\left(-i\omega \right)}^{n}}{n!}\int_{-\infty }^{+\infty }\frac{1}{\sqrt{2\pi }}e^{-\frac{x^{2}}{2}}x^{n}dx \end{array}$$ (16)

Let $E(x^{n})=\int_{-\infty }^{+\infty }\frac{1}{\sqrt{2\pi }}e^{-\frac{x^{2}}{2}}x^{n}dx$, where *x* ~ *N*(0, 1). Then, we have

$$E(x^{n})=\left\{\begin{array}{ll} 0, & n=2m+1\\ \frac{(2m)!}{2^{m}m}, & n=2m \end{array}\right..$$ (17)

Upon substituting Eq (17) into Eq (16), we have

$$\begin{array}{ll} \tilde{f}(\omega ) & =\underset{n\to +\infty }{\text{lim}}\int_{X}f(x)e^{-i\omega x}dx\\ & =\sum_{m=0}^{\infty }\frac{{\left(-i\omega \right)}^{2m}}{(2m)!}\frac{(2m)!}{2^{m}m!}\\ & =\sum_{m=0}^{\infty }\frac{1}{m!}{\left(-\frac{\omega^{2}}{2}\right)}^{m}\\ & =e^{-\frac{\omega^{2}}{2}}>0 \end{array}.$$

According to Theorem 3, the probability density function of the t distribution is the kernel function. □ In practice, generally, *n* ≥ 30.

**Corollary 1** When *n* = 1, the density function of the t distribution is

$$f(x)=\frac{1}{\pi (1+x^{2})}.$$ (18)

Then, Eq (18) is the kernel function.

Proof: Eq (18) is the Cauchy distribution density function, whose Fourier transform is [31]

$$\begin{array}{ll} f(x) & =\int_{-\infty }^{+\infty }e^{-i\omega x}\frac{1}{\left(1+x^{2}\right)}dx\\ & =e^{-\left|\omega \right|}>0 \end{array}.$$

Therefore, Eq (18) is the kernel function.

**Theorem 4** When *n* → +∞, the function

$$f(x)=\frac{\Gamma (\frac{n+1}{2})}{\sqrt{n\pi }\Gamma (\frac{n}{2})}{\left(1+\frac{\left|x\right|}{n}\right)}^{-\frac{n+1}{2}}$$ (19)

is the kernel function.

**Proof**: $\underset{n\to +\infty }{\text{lim}}f(x)=\underset{n\to +\infty }{\text{lim}}\frac{\Gamma (\frac{n+1}{2})}{\sqrt{n\pi }\Gamma (\frac{n}{2})}{\left(1+\frac{\left|x\right|}{n}\right)}^{-\frac{n+1}{2}}=\frac{1}{\sqrt{2\pi }}e^{-\frac{\left|x\right|}{2}}$, where $e^{-\frac{\left|x\right|}{2}}$ is the Laplace kernel function.

According to Theorem 3

$$\int_{-\infty }^{+\infty }e^{-\frac{\left|x\right|}{2}}e^{-i\omega x}dx\geq 0,$$

we have

$$\begin{array}{ll} \tilde{f}(\omega ) & =\underset{n\to +\infty }{\text{lim}}\int_{X}f(x)e^{-i\omega x}dx\\ & =\int_{X}\underset{n\to +\infty }{\text{lim}}f(x)e^{-i\omega x}dx\\ & =\int_{-\infty }^{+\infty }\frac{1}{\sqrt{2\pi }}e^{-\frac{\left|x\right|}{2}}e^{-i\omega x}dx\geq 0 \end{array}.$$

When *n* → ∞,

$$f(x)=\frac{\Gamma (\frac{n+1}{2})}{\sqrt{n\pi }\Gamma (\frac{n}{2})}{\left(1+\frac{\left|x\right|}{n}\right)}^{-\frac{n+1}{2}}$$

is the kernel function.

We call Eq (19) the pseudo t function.

**Corollary 2** When *n* = 1, the pseudo t function

$$f(x)=\frac{1}{\pi (1+\left|x\right|)}$$ (20)

is the kernel function.

Proof: According to Theorem 1, we just have to prove that $\int_{-\infty }^{+\infty }\frac{e^{-i\omega x}}{\pi (1+\left|x\right|)}dx\geq 0$.


$$\begin{array}{ll} & \int_{-\infty }^{+\infty }\frac{e^{-i\omega x}}{\pi (1+\left|x\right|)}dx\\ = & \int_{0}^{+\infty }\frac{e^{-i\omega x}}{\pi (1+x)}dx+\int_{-\infty }^{0}\frac{e^{-i\omega x}}{\pi (1-x)}dx\\ = & \int_{0}^{+\infty }\frac{\text{cos}(-\omega x)+i\;\text{sin}(-\omega x)}{\pi (1+x)}dx+\int_{-\infty }^{0}\frac{\text{cos}(-\omega x)+i\;\text{sin}(-\omega x)}{\pi (1-x)}dx \end{array}$$

Let *t* = −*x*. Then, we have

$$\begin{array}{ll} & \int_{-\infty }^{0}\frac{\text{cos}(-\omega x)+i\;\text{sin}(-\omega x)}{\pi (1-x)}dx\\ = & \int_{+\infty }^{0}\frac{\text{cos}(\omega t)+i\;\text{sin}(\omega t)}{\pi (1+t)}d(-t)\\ = & \int_{0}^{+\infty }\frac{\text{cos}(\omega t)+i\;\text{sin}(\omega t)}{\pi (1+t)}dt \end{array},$$

and

$$\begin{array}{ll} & \int_{-\infty }^{+\infty }\frac{e^{-i\omega x}}{\pi (1+\left|x\right|)}dx\\ = & \int_{0}^{+\infty }\frac{\text{cos}(-\omega x)+i\;\text{sin}(-\omega x)}{\pi (1+x)}dx+\int_{0}^{+\infty }\frac{\text{cos}(\omega t)+i\;\text{sin}(\omega t)}{\pi (1+t)}dt\\ = & \frac{2}{\pi }\int_{0}^{+\infty }\frac{\text{cos}(\omega x)}{1+x}dx \end{array}.$$

Now, the key problem is whether $\int_{0}^{+\infty }\frac{\text{cos}(\omega x)}{1+x}dx$ is positive or negative. We have

$$\begin{array}{ll} & \int_{0}^{+\infty }\frac{\text{cos}(\omega x)}{1+x}dx\\ = & \frac{1}{\omega }\int_{0}^{+\infty }\frac{1}{1+x}d\;\text{sin}(\omega x)\\ = & \frac{1}{\omega }(\frac{\text{sin}(\omega x)}{1+x}\left|{}_{0}^{+\infty }\right.-\int_{0}^{+\infty }\text{sin}(\omega x)d{\left(1+x\right)}^{-1})\\ = & \frac{1}{\omega }\int_{0}^{+\infty }\frac{\text{sin}(\omega x)}{{\left(1+x\right)}^{2}}dx\\ = & -\frac{1}{\omega^{2}}\int_{0}^{+\infty }\frac{1}{{\left(1+x\right)}^{2}}d\;\text{cos}(\omega x)\\ = & -\frac{1}{\omega^{2}}(\frac{\text{cos}(\omega x)}{{\left(1+x\right)}^{2}}\left|{}_{0}^{+\infty }\right.-\int_{0}^{+\infty }\text{sin}(\omega x)d{\left(1+x\right)}^{-2})\\ = & -\frac{1}{\omega^{2}}(-1+2\int_{0}^{+\infty }\frac{\text{cos}(\omega x)}{{\left(1+x\right)}^{3}}dx)\\ = & \frac{1}{\omega^{2}}-\frac{2}{\omega^{2}}\int_{0}^{+\infty }\frac{\text{cos}(\omega x)}{{\left(1+x\right)}^{3}}dx \end{array}.$$

Because $\int_{0}^{+\infty }\frac{\text{cos}(\omega x)}{{\left(1+x\right)}^{3}}dx\leq \int_{0}^{+\infty }\frac{1}{{\left(1+x\right)}^{3}}dx=\frac{1}{2}$, we can determine that

$$\begin{array}{ll} & \frac{1}{\omega^{2}}-\frac{2}{\omega^{2}}\int_{0}^{+\infty }\frac{\text{cos}(\omega x)}{{\left(1+x\right)}^{3}}dx\\ = & \frac{1}{\omega^{2}}(1-2\int_{0}^{+\infty }\frac{\text{cos}(\omega x)}{{\left(1+x\right)}^{3}}dx)\geq 0 \end{array}.$$

Because

$$\int_{0}^{+\infty }\frac{\text{cos}(\omega x)}{1+x}dx\geq 0,$$

we have

$$\int_{-\infty }^{+\infty }\frac{e^{-i\omega x}}{\pi (1+\left|x\right|)}dx\geq 0.$$

Therefore, $f(x)=\frac{1}{\pi (1+\left|x\right|)}$ is the kernel function.□

The kernel function in Corollary 2 $f(x)=\frac{1}{\pi (1+\left|x\right|)}$ can be generalized as $f(x)=\frac{1}{c(1+\left|x\right|)}$. There is the following Corollary.

**Corollary 3 When** *c* > 0, the function

$$f(x)=\frac{1}{c(1+\left|x\right|)}$$ (21)

is the kernel function.

The constant *c* in the above equation can be regarded as the scale parameter, so the kernel function in Corollary 3 is a multi-scale kernel function, which can adapt to the samples with drastic changes when the scale parameter is small, and can adapt to the samples with gentle changes when the scale parameter is large. The figure of multi-scale t kernel function with different parameters is as follow.

**Fig 1:**
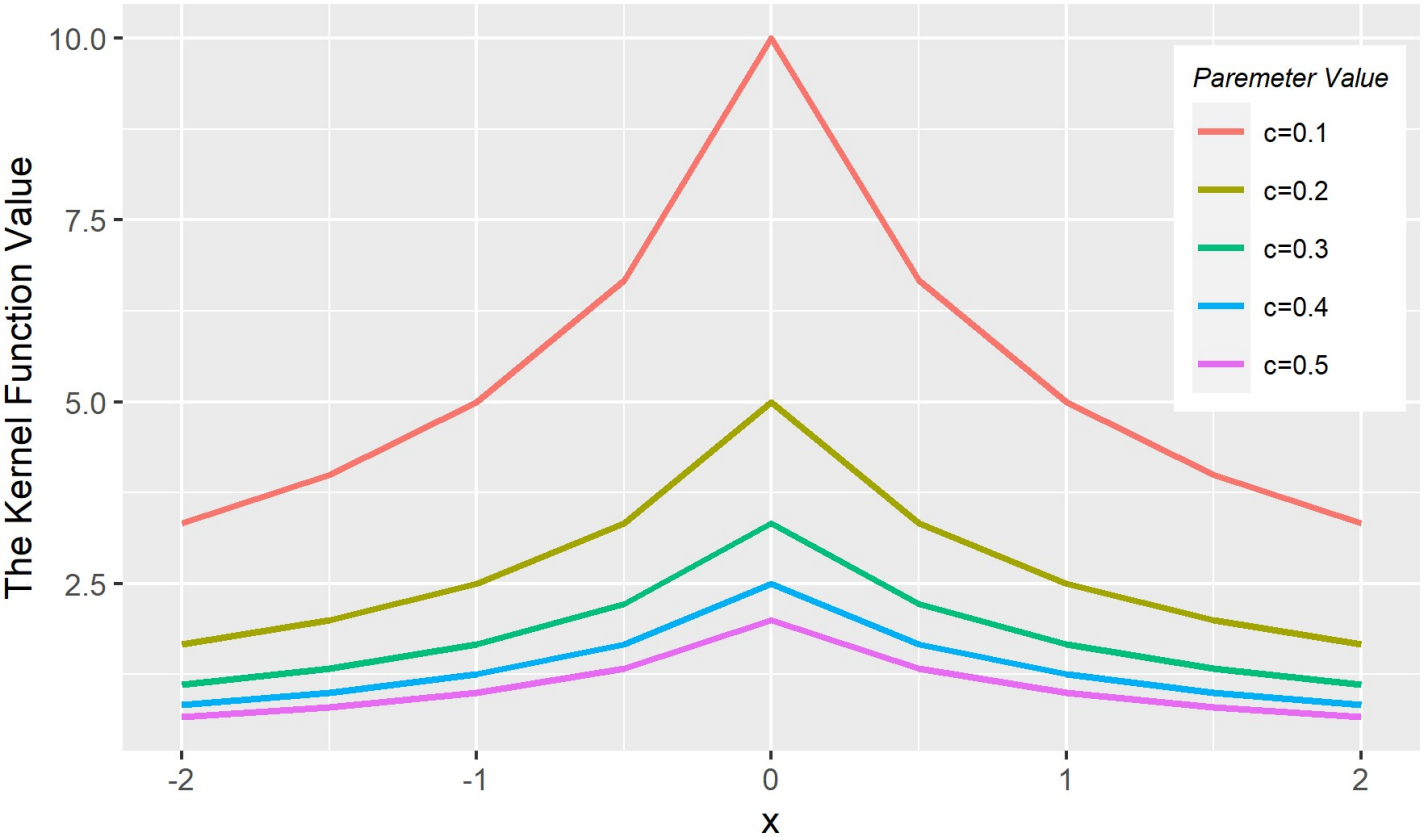
Multi-scale t kernel function under different parameters.

It can be seen from the Fig 1 that the kernel function gradually flattens with the increase of scale parameters. The multi-scale t-class kernel function constructed in Corollary 3 has rich scale choices, which makes it have better adaptability when processing complex data.

If only the traditional kernel functions such as polynomial kernel and hyperbolic tangent kernel are combined linearly, there is no basis for the selection and combination of kernel function parameters, and the uneven distribution of samples still cannot be solved satisfactorically, which limits the expression ability of decision function. The t-class kernel functions constructed by us can be generalized to multi-scale functions eventually. With the gradual maturity and improvement of wavelet theory and multi-scale analysis theory, the multi-scale kernel method has a good theoretical background by introducing scale space.

Some t-class kernel functions are constructed in this section, and they can be part of the weighted kernel function. By the experimental analysis in Section 4, the t-class kernel function can reduce the dimensionality of gene expression data effectively and improve the classification performance of subsequent machine learning methods.

### 3.3 WKPCA dimension reduction algorithm

**Fig 2:**
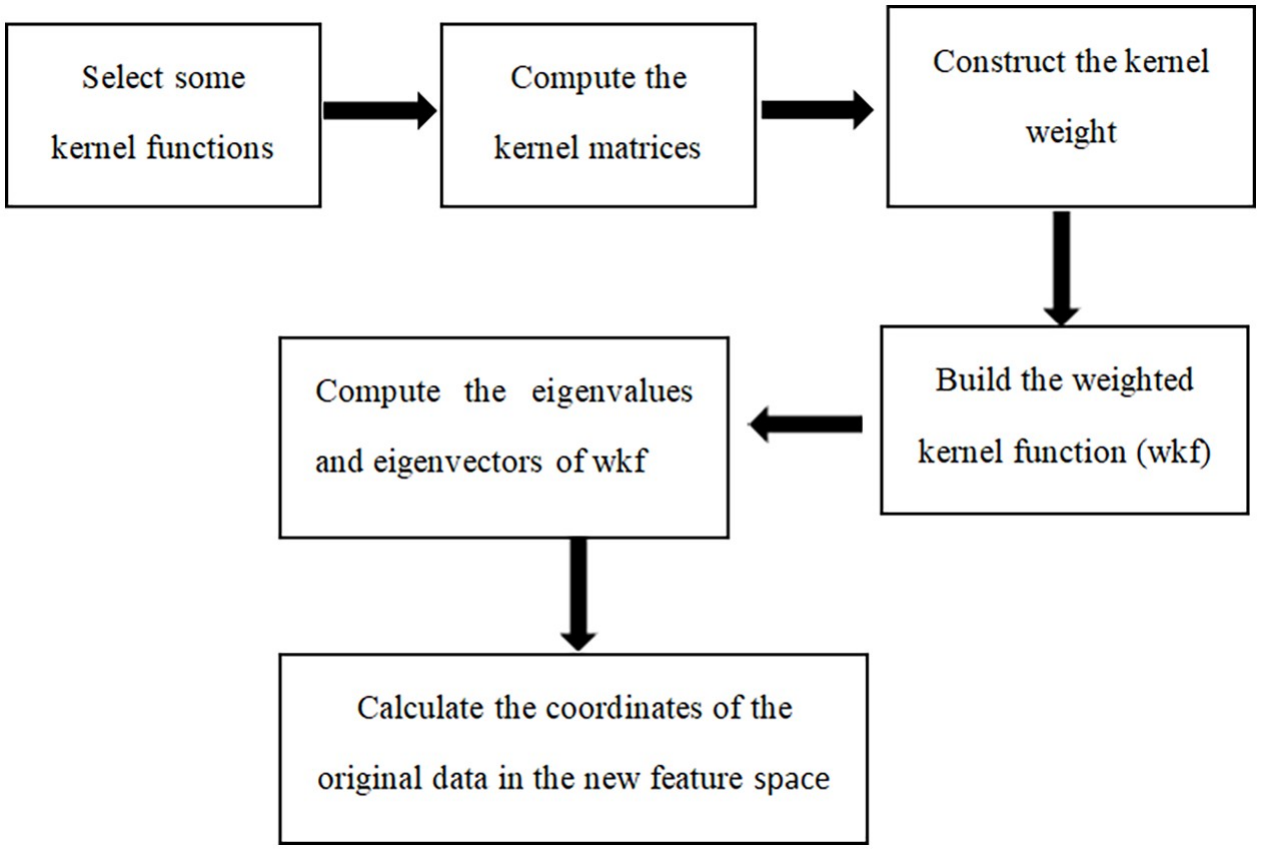
The frame of the WKPCA dimension reduction algorithm.

According to the theory of kernel principal component analysis and weighted kernel function construction, the basic framework of the WKPCA dimension reduction algorithm is shown in Fig 2.

#### 3.3.1 WKPCA dimension reduction algorithm design

Obviously, the kernel principal component depends on the selection of the kernel function. When constructing the weighted kernel function to reduce the dimensionality, kernel functions such as the Gaussian kernel, Laplace kernel, hyperbolic tangent kernel and polynomial kernel functions are generally selected. We can also choose the t-class kernel function, which is constructed in Section 3.2. Since the weighted kernel principal component requires calculating the eigenvalues and eigenvectors of the weighted kernel matrix, first, the corresponding weighted kernel matrix should be computed using the training samples.


$$Κ(x_{i},x_{j})=\sum_{s=1}^{n}\omega_{s}{Κ}_{s}(x_{i},x_{j})={\left(\sum_{s=1}^{n}\omega_{s}\kappa_{s}(x_{i},x_{j})\right)}_{m\times m}.$$ (22)

According to Eq (22), if the sample size is only a few hundred samples, for example, *m* = 400, then the kernel matrix will contain 160,000 data points. With the increase of the sample size, the time cost of calculating the weighted kernel matrix will greatly increase.

To improve the operational efficiency of the algorithm, the following methods can be adopted. The Gaussian kernel and t-class kernel functions can be regarded as the distance function of any two samples, while the hyperbolic tangent kernel and polynomial kernel functions can be regarded as the function of the inner product of any two samples. Take the pseudo-t kernel function when n = 1 as an example,

$$\kappa (x_{i},x_{j})=\frac{1}{\pi (1+\left|x_{i}-x_{j}\right|)}.$$ (23)

Its kernel matrix is

$$\begin{array}{ll} Κ(x_{i},x_{j}) & ={\left(\frac{1}{\pi (1+\left|x_{i}-x_{j}\right|)}\right)}_{m\times m}\\ & ={\left(\frac{1}{\pi (1+\left|dist_{ij}\right|)}\right)}_{m\times m} \end{array},$$ (24)

where *dist*_*ij*_ = ‖*x*_*i*_ − *x*_*j*_‖ is the Euclidean distance of any two samples and *Dist* = (*dist*_*ij*_)_*m*×*m*_ is the distance matrix of the sample set.

Let *M* = (*x*_*ij*_)_*m*×*n*_, and define the matrix function as

$$g(M)={\left(g(x_{ij})\right)}_{m\times n}$$ (25)

According to Eqs (24) and (25), the kernel matrix based on the pseudo-t kernel can be regarded as a function of the distance matrix, i.e.,

$$Κ(x_{i},x_{j})=Κ(Dist)={\left(\kappa (\left|x_{i}-x_{j}\right|)\right)}_{m\times m}.$$ (26)

Similarly, the kernel matrix based on the hyperbolic tangent kernel and polynomial kernel can be regarded as a function of the inner product matrix, i.e.,

$$DD^{T}={\left(x_{i}^{T}x_{j}\right)}_{m\times m}$$

$$Κ(x_{i},x_{j})=Κ(DD^{T})={\left(\kappa (x_{i}^{T}x_{j})\right)}_{m\times m}$$ (27)

Therefore, the distance matrix and the inner product matrix can be substituted into the kernel function as a whole to get the corresponding "kernel matrix". The above process is called the vectorized computation method. In terms of algorithm design, vectorization is faster and more efficient than the multiple loop statements shown.

The dimensional reduction algorithm flow of WKPCA is given as shown in Table 1.

**Table 1:** WKPCA dimension reduction algorithm flow.

Algorithm: WKPCA Dimension Reduction
Inputs: *D* = {*x*_*i*_, *i* = 1, 2, …, *m*}: Training sample set; *p*: Feature number *Dist* = (*dist*_*ij*_)_*m*×*m*_: Distance matrix; $DD^{T}={\left(x_{i}^{T}x_{j}\right)}_{m\times m}$: Inner product matrix
Output: *z* = {*z*_*i*_, *i* = 1, 2, …, *m*}: The projection of the sample set D in the new feature space.
1. Definite the kernel function *κ*_*s*_(*x*, *y*), *s* = 1, 2, …, *q*
2. Compute the distance matrix *Dist* and inner product matrix *DD*^*T*^
3. Compute the kernel matrix corresponding to the *s*^th^ kernel function ${Κ}_{s}(x_{i},x_{j})={Κ}_{s}(Dist)={\left(\kappa_{s}(\left\|x_{i}-x_{j}\right\|)\right)}_{m\times m},{Κ}_{s}(x_{i},x_{j})={Κ}_{s}(DD^{T})={\left(\kappa_{s}(x_{i}^{T}x_{j})\right)}_{m\times m}$
4. Compute the eigenvalues $\lambda_{j}^{s},j=1,2,\ldots ,m$ of K_*s*_(*x*, *y*)
5. for d in 1:p
6. for s in 1:q
7. Calculate the weight of the kernel matrix $\omega_{s}=\frac{\sum_{j=1}^{d}\lambda_{j}^{s}}{\sum_{s=1}^{q}\sum_{j=1}^{d}\lambda_{j}^{s}}$
8. end for
9. Definite the weight kernel function $\kappa (x,y)=\sum_{s=1}^{q}\omega_{s}\kappa_{s}(x,y)$
10. Compute the kernel matrix of *κ*(*x*, *y*) $Κ(x_{i},x_{j})=\sum_{s=1}^{q}\omega_{s}{Κ}_{s}(x_{i},x_{j})={\left(\sum_{s=1}^{q}\omega_{s}\kappa_{s}(x_{i},x_{j})\right)}_{m\times m}$
11. Compute the eigenvalues and eigenvectors *λ*_*j*_ and $\alpha^{j}=(\alpha_{1}^{j},\alpha_{2}^{j},\ldots ,\alpha_{m}^{j}{)}^{\prime }$ of K(*x*, *y*), *j* = 1, 2, …, *m*
12. Compute sample *x* the *j*^th^ component in the new coordinate system $z^{j}=\sum_{i=1}^{m}\alpha_{i}^{j}\phi^{T}(x_{i})\phi (x)=\sum_{i=1}^{m}\alpha_{i}^{j}\kappa (x_{i},x),j=1,2,\ldots ,d$
13. end for

First, the input of the WKPCA algorithm includes 3 to 4 parts—the original data matrix *D* = (*x*_*ij*_)_*m*×*p*_, the number *p* of features contained in the data, and the distance matrix or inner product matrix corresponding to the original data set *D*. If we define both the t-class kernel (or Laplace kernel) and the hyperbolic tangent kernel (or polynomial sum) in step 1 of the algorithm, we need to use both the distance matrix and the inner product matrix; otherwise, only one type of matrix will be input.

For the first line of the algorithm, in order to ensure the simplicity of the algorithm, two or three kernel functions are generally defined. Based on the distance matrix and inner product matrix, the kernel matrix and its eigenvalues are computed from Lines 2 to 4. In Line 5, *d* represents the selected dimension after feature reduction, where *d* < *p*. The weight of each kernel function is determined between Line 6 and 8. The kernel matrix of the weighted kernel function and its eigenvalues and eigenvectors are calculated between Lines 9 and 11. The *d* dimensional coordinates of all the samples in the new feature space are calculated in Line 12.

Time complexity analysis: Due to vectorized computation, the time used to calculate the distance and inner product matrix in Line 2 is *O*(1), and the time used in Lines 1 to 4 is *O*(*q*). The time consumption of the WKPCA algorithm mainly occurs in Lines 5 to 13, and its time complexity is *O*((*m* + *q*)*p*). Since the number *q* of kernel functions is much smaller than the sample size *m*, the total time complexity of this algorithm is *O*(*mp*). It is important to point out that in general, *m* > *p* or *m* >> *p*, but for some data sets, such as the gene expression data set, *m* ≪ *p*. Through the experimental analysis in Section 4, it can be concluded that after the dimension reduction of the WKPCA, the value of *p* only needs to be a few percent of the total number of variables to achieve a better classification prediction effect, and the time cost is moderate.

## 4 Experimental results and analysis

In this section, the t-class kernel functions constructed in Section 3.2 are weighted and combined. WKPCA dimension reduction is performed on 6 real gene expression data sets based on the t-class weighted kernel function to obtain unrelated principal components. According to Eq (14), the number *d* of principal components retained is determined. Then, the current mainstream machine learning methods including naive Bayes (NB) [32], support vector machines (SVM) [33], k-nearest neighbour (KNN) [34], random forest (RF) [35], and iterative random forest (IRF) [36–38] are used to make classification predictions for the subset after dimension reduction. The above machine learning algorithm is used to perform classification prediction on the all-variable (AV) data set, and the data subsets of linear principal component analysis (PCA) dimension reduction, single kernel principal component (SKPCA) dimension reduction and weighted kernel principal component analysis (WKPCA) dimension reduction.

### 4.1 Experimental design

The experiments were conducted on a machine equipped with the Windows 10 64-bit operating system, an Intel i7-10510 μ 2.3 GHz CPU and 16 GB memory. The algorithm was implemented in the R language (R 3.6.3). The 6 real data sets used in this paper are from the Broad Institute Genome Data Analysis Center (http://portals.broadinstitute.org/cgi-bin/cancer/datasets.cgi). See Table 2 for detailed information.

**Table 2:** Data information.

Data Name	Observations	Features	Categories
Breast	98	1213	3
DLBCL-B	180	661	3
DLBCL-D	129	3795	4
Leukaemia	248	985	6
Multi-A	103	5565	4
Lung	197	1000	4

To compare the performances of the machine learning classification algorithms in different dimensions, the classification macro accuracy, macro precision, macro recall, macro *F*1 are used and their specific definitions are as follows.

Suppose that the data set *D* has *k* categories. The *i*^th^ category is considered as a positive class, and the remaining *k* − 1 categories are deemed to be negative class. We use *P*_*i*_, *R*_*i*_, *F*1_i_ to denote the precision, recall and of *i*^th^ category respectively.


$$MacroP=\frac{1}{k}\sum_{i=1}^{k}P_{i}$$ (28)

$$MacroR=\frac{1}{k}\sum_{i=1}^{k}R_{i}$$ (29)

$$MacroF1=\frac{1}{k}\sum_{i=1}^{k}F1_{i}$$ (30)

From Eqs (28) to (30), it can be seen that the so-called macro is to calculate the precision, recall rate and *F*1 of each category, and calculate their average value respectively, so as to evaluate the performance of the algorithm in multi-class problems. The larger the macro precision, macro recall and macro F1, the better the performance of the algorithm. AUC value of the area under the ROC curve is also used in the evaluation criteria [39].

Since the number of categories of the 6 datasets is more than 2, the definition of AUC for multi-classification problems given by Hand and Till [40] is adopted. Nonlinear SVM based on Gaussian kernel function is used. The parameters of the SVM and KNN classification methods are realized by the machine learning adjustable parameter functions tune.svm and tune.kknn in the R language [41]. In the tune.svm, the parameter grid search range is set to 0.1 to 4 at step length 0.1. In the tune.kknn, the parameter grid search range is set to 1 to 30 at step length 1. RF is set to 500 trees by default, and the number of IRF iterations is set to 6.

To evaluate the overall classification performance of WKPCA combined with various machine learning algorithms, the definition of the optimal performance rate (OPR) of WKPCA is given in this paper.

$$OPR=\frac{PN}{MN\times DN\times EN},$$ (31)

where *MN* is the number of machine learning algorithms, *DN* is the number of data sets, *EN* is the number of evaluation indexes, and *PN* is the number of WKPCA dimension reduction algorithms reaching the maximum under each evaluation index.

By extending Eq (31), the cumulative optimal performance rate (COPR) of WKPCA is given

$$COPR=\frac{\sum_{i=1}^{s+1}PN_{i}}{MN\times DN\times EN},$$ (32)

where *PN*_*i*_ is the number of WKPCA dimension reduction algorithms reaching the *j*^th^ maximum under each evaluation index and *s* is the number of methods compared with WKPCA.

### 4.2 Comparison experiment

Based on the t-class weighted kernel function, WKPCA dimension reduction is performed for the 6 gene expression data sets in Table 2. Through a large number of comparative experiments, for different datasets and different classification methods, Different kernel combination formulas for dimensionality reduction will result in different classification performance. In order to achieve the relatively optimal performance of the classification algorithm after dimensionality reduction of kernel principal component, the following three forms of kernel combination formula are mainly adopted.


$$\kappa (x_{i},x_{j})=\omega_{1}\frac{1}{c_{1}(1+{\left\|x_{i}-x_{j}\right\|}^{2})}+\omega_{2}\frac{1}{c_{2}(1+\left\|x_{i}-x_{j}\right\|)}$$ (33)

$$\kappa (x_{i},x_{j})=\omega_{1}\text{exp}(-\gamma {\left\|x_{i}-x_{j}\right\|}^{2})+\omega_{2}\frac{1}{c_{1}(1+{\left\|x_{i}-x_{j}\right\|}^{2})}+\omega_{3}\frac{1}{c_{2}(1+\left\|x_{i}-x_{j}\right\|)}$$ (34)

$$\kappa (x_{i},x_{j})=\omega_{1}\text{exp}(-\gamma \left\|x_{i}-x_{j}\right\|)+\omega_{2}\frac{1}{c_{1}(1+{\left\|x_{i}-x_{j}\right\|}^{2})}+\omega_{3}\frac{1}{c_{2}(1+\left\|x_{i}-x_{j}\right\|)}$$ (35)

The above equations are used to reduce dimension of the original data set based on the kernel principal component, and compared with the traditional Gaussian kernel, the experimental results are shown in Tables 3 to 8 in this paper.

**Table 3:** Performance measurement comparisons of machine learning methods based on the Breast data set after WKPCA dimension reduction.

Method Name	Number of Features	Accuracy	Macro-Recall	Macro-Precision	Macro-*F*1
NB_AV	1213	0.9088	0.8291	0.9179	0.8357
NB_PCA	16	0.7500	0.8456	0.8790	0.8609
NB_SKPCA_(36)	16	0.7449	0.7597	0.7113	0.6937
NB_WKPCA_(33)	16	**0.9290**	**0.8874**	**0.9247**	**0.8921**
SVM_AV	1213	0.5200	0.6325	0.5200	0.6797
SVM_PCA	14	0.8679	0.7244	0.8965	0.8426
SVM_SKPCA_(36)	14	0.8984	0.8250	0.9180	0.8472
SVM_WKPCA_(33)	14	**0.9184**	**0.8394**	**0.9375**	**0.8625**
KNN_AV	1213	0.8876	0.7731	0.8887	0.8052
KNN_PCA	14	0.9085	0.8287	0.9211	0.8355
KNN_SKPCA_(36)	14	0.8980	0.8041	0.8754	0.8253
KNN_WKPCA_(33)	14	**0.9290**	**0.8847**	**0.9348**	**0.8955**
RF_AV	1213	**0.8673**	0.6889	0.7951	0.6020
RF_PCA	15	0.8571	0.6960	0.8051	0.6888
RF_SKPCA_(36)	15	0.8571	0.6990	0.8070	0.6911
RF_WKPCA_(33)	15	**0.8673**	**0.7043**	**0.8215**	**0.7018**
IRF_AV	1213	**0.9085**	**0.7937**	**0.9112**	**0.8225**
IRF_PCA	6	0.8374	0.6866	0.8052	0.6970
IRF_SKPCA_(36)	6	0.8984	0.8050	0.8165	0.8060
IRF_WKPCA_(33)	6	0.8979	0.7828	0.8990	0.8125

**Table 4:** Performance measurement comparisons of machine learning methods based on the DLBCL-B data set after WKPCA dimension reduction.

Method Name	Number of Features	Accuracy	Macro-Recall	Macro-Precision	Macro-*F*1
NB_AV	661	**0.9444**	**0.9342**	**0.9395**	**0.9327**
NB_PCA	8	0.8444	0.8356	0.8337	0.8297
NB_SKPCA_(36)	8	0.4111	0.3469	0.3849	0.3726
NB_WKPCA_(33)	8	0.9333	0.9220	0.9303	0.9243
SVM_AV	661	0.4833	0.3333	0.3786	0.3460
SVM_PCA	4	**0.9611**	0.9543	**0.9563**	**0.9543**
SVM_SKPCA_(36)	4	0.5000	0.3592	0.3862	0.3640
SVM_WKPCA_(33)	4	0.9556	**0.9561**	0.9485	0.9507
KNN_AV	661	0.8500	0.8168	0.8610	0.8189
KNN_PCA	5	**0.9556**	**0.9498**	**0.9481**	**0.9480**
KNN_SKPCA_(36)	5	0.7611	0.7400	0.7412	0.7201
KNN_WKPCA_(33)	5	0.9444	0.9377	0.9368	0.9358
RF_AV	661	0.3833	0.9080	0.9290	0.9143
RF_PCA	6	0.9167	0.8989	0.9162	0.9008
RF_SKPCA_(36)	6	0.7667	0.7086	0.7302	0.7011
RF_WKPCA_(34)	6	**0.9500**	**0.9447**	**0.9425**	**0.9397**
IRF_AV	661	0.8944	0.8717	0.8918	0.8780
IRF_PCA	5	0.9167	**0.9024**	**0.9121**	**0.9004**
IRF_SKPCA_(36)	5	0.7778	0.7331	0.7432	0.7262
IRF_WKPCA_(33)	5	**0.9722**	0.8877	0.8838	0.8806

**Table 5:** Performance measurement comparisons of machine learning methods based on the DLBCL-D data set after WKPCA dimension reduction.

Method Name	Number of Features	Accuracy	Macro-Recall	Macro-Precision	Macro-*F*1
NB_AV	3795	NA	NA	NA	NA
NB_PCA	9	0.6828	0.7033	0.6881	0.6646
NB_SKPCA_(36)	9	0.2791	0.3548	0.3933	0.3660
NB_WKPCA_(34)	9	**0.7683**	**0.7811**	**0.7543**	**0.7529**
SVM_AV	3795	0.3803	0.2500	0.3021	0.3720
SVM_PCA	9	0.7142	0.6932	0.7500	0.6833
SVM_SKPCA_(36)	9	0.4191	0.3285	0.3570	0.3430
SVM_WKPCA_(34)	9	**0.8071**	**0.7978**	**0.8251**	**0.7859**
KNN_AV	3795	0.6822	0.6119	**0.7135**	0.6131
KNN_PCA	10	0.6514	0.6052	0.6533	0.6042
KNN_SKPCA_(36)	10	0.2954	0.2476	0.2667	0.2665
KNN_WKPCA_(33)	10	**0.7054**	**0.6664**	0.6989	**0.6674**
RF_AV	3795	0.7364	0.6516	0.7204	0.6670
RF_PCA	17	0.7287	0.6641	0.7183	0.6627
RF_SKPCA_(36)	17	0.5504	0.4872	0.5815	0.4862
RF_WKPCA_(34)	17	**0.7442**	**0.7004**	**0.7579**	**0.7062**
IRF_AV	3795	**0.7683**	**0.7359**	**0.7694**	**0.7271**
IRF_PCA	9	0.6840	0.6685	0.7080	0.6600
IRF_SKPCA_(36)	9	0.4585	0.3678	0.3968	0.3864
IRF_WKPCA_(33)	9	0.7286	0.6978	0.6986	0.6812

**Table 6:** Performance measurement comparisons of machine learning methods based on the Leukaemia data set after WKPCA dimension reduction.

0.Method Name	Number of Features	Accuracy	Macro-Recall	Macro-Precision	Macro-*F*1
NB_AV	985	0.9758	0.9458	0.9537	0.9476
NB_PCA	7	**0.9799**	**0.9637**	**0.9712**	**0.9616**
NB_SKPCA_(36)	7	0.3993	0.2675	0.3070	0.2840
NB_WKPCA_(33)	7	0.9677	0.9372	0.9646	0.9440
SVM_AV	985	0.3188	0.1667	0.1826	0.1750
SVM_PCA	11	**0.9838**	**0.9597**	**0.9866**	**0.9682**
SVM_SKPCA_(36)	11	0.8509	0.7158	0.7540	0.7225
SVM_WKPCA_(35)	11	0.9758	0.9215	0.9540	0.9350
KNN_AV	985	0.9477	0.9000	0.9048	0.8779
KNN_PCA	9	0.9759	0.9519	0.9467	0.9480
KNN_SKPCA_(36)	9	0.7903	0.6487	0.6850	0.6560
KNN_WKPCA_(33)	9	**0.9838**	**0.9597**	**0.9813**	**0.9652**
RF_AV	985	0.9718	0.9250	0.9450	0.9359
RF_PCA	19	**0.9878**	**0.9722**	0.9837	**0.9729**
RF_SKPCA_(36)	19	0.2943	0.1667	0.2040	0.1890
RF_WKPCA_(35)	19	0.9637	0.9120	**0.9850**	0.9720
IRF_AV	985	0.9516	0.9130	0.9197	0.9138
IRF_PCA	26	0.7683	0.9469	**0.9814**	**0.9633**
IRF_SKPCA_(36)	26	0.8344	0.7579	0.8075	0.7889
IRF_WKPCA_(35)	26	**0.9717**	**0.9510**	0.9565	0.9466

**Table 7:** Performance measurement comparisons of machine learning methods based on the Multi-A data set after WKPCA dimension reduction.

Method Name	Number of Features	Accuracy	Macro-Recall	Macro-Precision	Macro-*F*1
NB_AV	5565	NA	NA	NA	NA
NB_PCA	10	0.9214	**0.9324**	0.9303	0.9206
NB_SKPCA_(36)	10	0.4743	0.4508	0.4825	0.4670
NB_WKPCA_(35)	10	**0.9224**	0.9229	**0.9305**	**0.9227**
SVM_AV	5565	0.1933	0.2655	0.3045	0.2880
SVM_PCA	14	0.9800	**0.9875**	**0.9800**	**0.9804**
SVM_SKPCA_(36)	14	0.3105	0.3942	0.4120	0.4050
SVM_WKPCA_(35)	14	**0.9805**	0.9838	0.9775	0.9784
KNN_AV	5565	0.9519	0.9557	0.9442	0.9455
KNN_PCA	9	0.9510	0.9546	0.9529	0.9454
KNN_SKPCA_(36)	9	0.1743	0.2500	0.2656	0.2765
KNN_WKPCA_(33)	9	**0.9705**	**0.9775**	**0.9700**	**0.9692**
RF_AV	5565	0.9705	0.9775	0.9700	0.9692
RF_PCA	29	0.9800	0.9875	**0.9800**	**0.9804**
RF_SKPCA_(36)	29	0.7948	0.8039	0.7952	0.7789
RF_WKPCA_(33)	29	**0.9805**	**0.9882**	0.9750	0.9794
IRF_AV	5565	**0.9705**	**0.9754**	**0.9700**	**0.9685**
IRF_PCA	17	0.9414	0.9408	0.9538	0.9379
IRF_SKPCA_(36)	17	0.7648	0.7734	0.7728	0.7490
IRF_WKPCA_(35)	17	0.9419	0.9497	0.9388	0.9392

**Table 8:** Performance measurement comparisons of machine learning methods based on the Lung data set after WKPCA dimension reduction.

Method Name	Number of Features	Accuracy	Macro-Recall	Macro-Precision	Macro-*F*1
NB_AV	1001	0.9645	**0.9668**	0.9454	0.9518
NB_PCA	4	0.9645	0.9519	0.9616	0.9526
NB_SKPCA_(36)	4	0.7922	0.8049	0.7429	0.7527
NB_WKPCA_(35)	4	**0.9696**	0.9623	**0.9635**	**0.9590**
SVM_AV	1001	0.7055	0.2500	0.2890	0.2660
SVM_PCA	**8**	0.9746	0.9703	0.9693	**0.9675**
SVM_SKPCA_(36)	**8**	0.7208	0.3168	0.3430	0.3245
SVM_WKPCA_(35)	**8**	**0.9796**	**0.9721**	**0.9767**	0.9728
KNN_AV	1001	0.9392	0.8849	0.9394	0.9056
KNN_PCA	7	0.9644	0.9417	0.9644	0.9502
KNN_SKPCA_(36)	7	0.8779	0.8204	0.8150	0.7988
KNN_WKPCA_(33)	7	**0.9695**	**0.9536**	**0.9677**	**0.9574**
RF_AV	1001	0.9542	0.8997	**0.9670**	0.9201
RF_PCA	7	0.9594	0.9400	0.9583	0.9453
RF_SKPCA_(36)	7	0.8374	0.6857	0.6717	0.6467
RF_WKPCA_(33)	7	**0.9645**	**0.9519**	0.9616	**0.9526**
IRF_AV	1001	0.9390	0.8882	0.9400	0.9102
IRF_PCA	10	0.9593	0.9399	0.9554	0.9444
IRF_SKPCA_(36)	10	0.9389	0.8756	0.9411	0.8965
IRF_WKPCA_(35)	10	**0.9645**	**0.9519**	**0.9616**	**0.9526**

For SKPCA dimension reduction, the selected single kernel function is the Gaussian kernel

$$\kappa (x_{i},x_{j})=\text{exp}(-\gamma {\left\|x_{i}-x_{j}\right\|}^{2})$$ (36)

The weights in the Eqs (29), (30) and (31) are determined according to the Eq (12) in the paper. For the determination of scale parameters *c*_1_, *c*_2_, and *γ*, the wrapper learning algorithm is used. The parameter selection of the kernel function is combined with the subsequent machine learning classification algorithm, and the parameters that make the classification performance optimal are selected through cross validation. Finally, these parameters are set to *c*_1_ = 0.1, *c*_2_ = 0.2 and *γ* = 0.1.

According to the experimental results in Tables 3 to 8, we can find that it is not difficult to find the relatively optimal parameters.

The above five machine learning methods are used to classify and predict the following 4 data sets: (1) one with all variables; (2) one obtained by linear principal component analysis dimension reduction; (3) one obtained by single kernel function dimension reduction; and (4) the last obtained by weighted kernel function dimension reduction. The comparison results obtained through nested 5-fold cross validation are shown in Tables 3 to 8, in which the optimal performance index values are bolded.

We combine the five machine learning methods with AV, PCA, SKCPCA and WKPCA, so each table (Table 3 through Table 8) contains 20 methods. In these six tables, the machine learning classification algorithm combined with WKPCA corresponds to the best performance. Taking the Breast data set as an example, compared with AV, PCA and SKPCA, NB_WKPCA, SVM_WKPCA, KNN_WKPCA and RF_WKPCA were all the largest in the four evaluation indexes. However, IRF_WKPCA did not reach the maximum on four evaluation indexes, and the other tables showed similar results. According to the experimental results from Table 3 through Table 8, among the 5 machine learning methods combined with WKPCA, there are 4, 14, 5, 13, 10 and 3 that do not reach the maximum on the four evaluation indexes. Therefore, according to Eq (31), the optimal performance rate of WKPCA on these 6 data sets is

$$OPR=\frac{120-(4+14+5+13+10+3)}{5\times 6\times 4}=\frac{71}{120}\approx 0.5917.$$

According to Eq (32), the cumulative optimal performance rate of WKPCA on these 6 data sets is

$$COPR=\frac{77+37}{120}\approx 0.95$$

Through OPR and COPR values, it can be concluded that the WKPCA algorithm is optimal at 71 and suboptimal at 37, and the cumulative optimal performance rate of the first two positions reaches 95%. This indicates that WKPCA dimension reduction can effectively improve the classification performance of the current mainstream machine learning algorithms. In other words, WKPCA is superior to AV, PCA and SKPCA in most cases.

It should be noted that for the SVM classification algorithm, if all variables are involved in the modelling without dimension reduction, the classification accuracy of SVM_AV on the 6 data sets is only 0.5200, 0.4833, 0.3803, 0.3188, 0.1933 and 0.7053. After WKPCA dimension reduction, the SVM classification accuracy was greatly improved, reaching 0.9184, 0.9556, 0.8071, 0.9758, 0.9805 and 0.9796, respectively. It is shown that when the number of features in the data set is much larger than the number of samples, the classification performance of some algorithms will be degraded or even become invalid if all variables are involved in the model. However, after WKPCA dimension reduction, a few principal components unrelated to each other are retained, redundant information (noise interference) is eliminated and the main information related to the sample category is retained, which improves the classification performance of the machine learning algorithm. In Tables 5 and 7, NB_AV has missing values (NA) on four performance indexes. According to experimental analysis, the reason for this problem is that the sample variance is 0 for at least 1 column variable. If all variables are included in the NB model for classification, this algorithm will fail. However, after the dimension reduction of WKPCA, PCA and SKPCA, the zero variance can be avoided, and normal classification results can be obtained.

**Fig 3:**
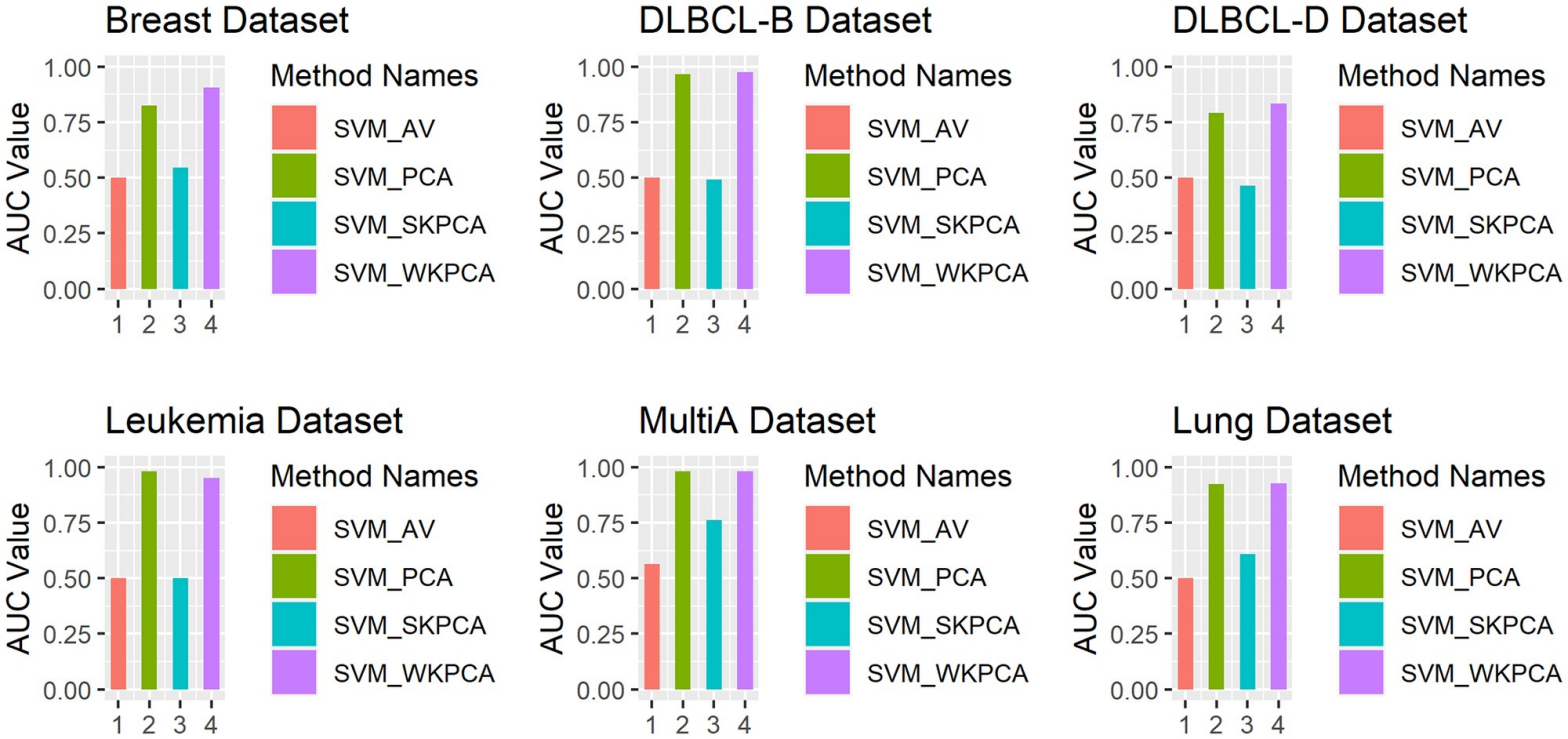
Comparison of the SVM_AV, SVM_PCA, SVM_SKPCA and SVM_WKPCA method AUC values.

**Fig 4:**
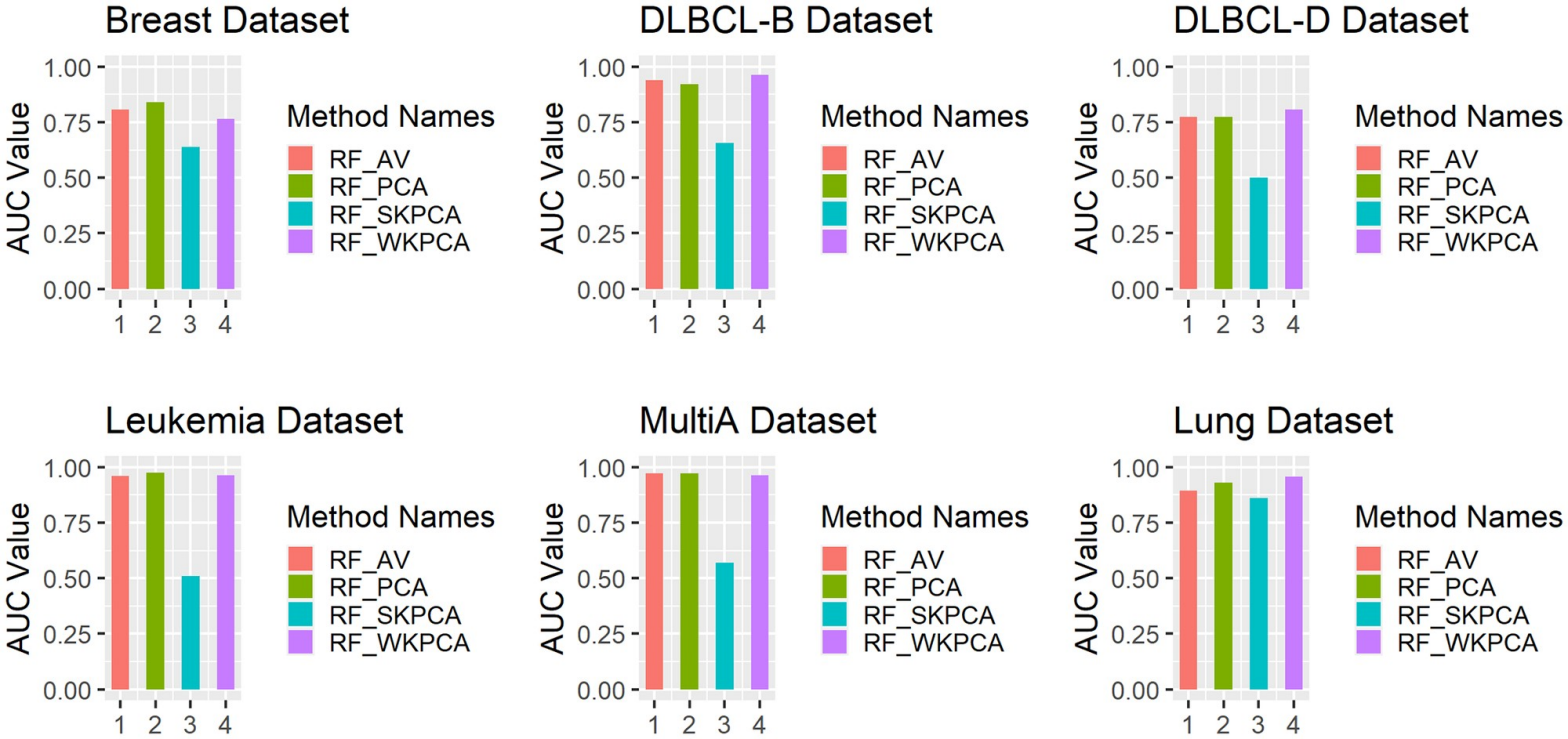
Comparison of the RF_AV, RF_PCA, RF_SKPCA and RF_WKPCA method AUC values.

**Fig 5:**
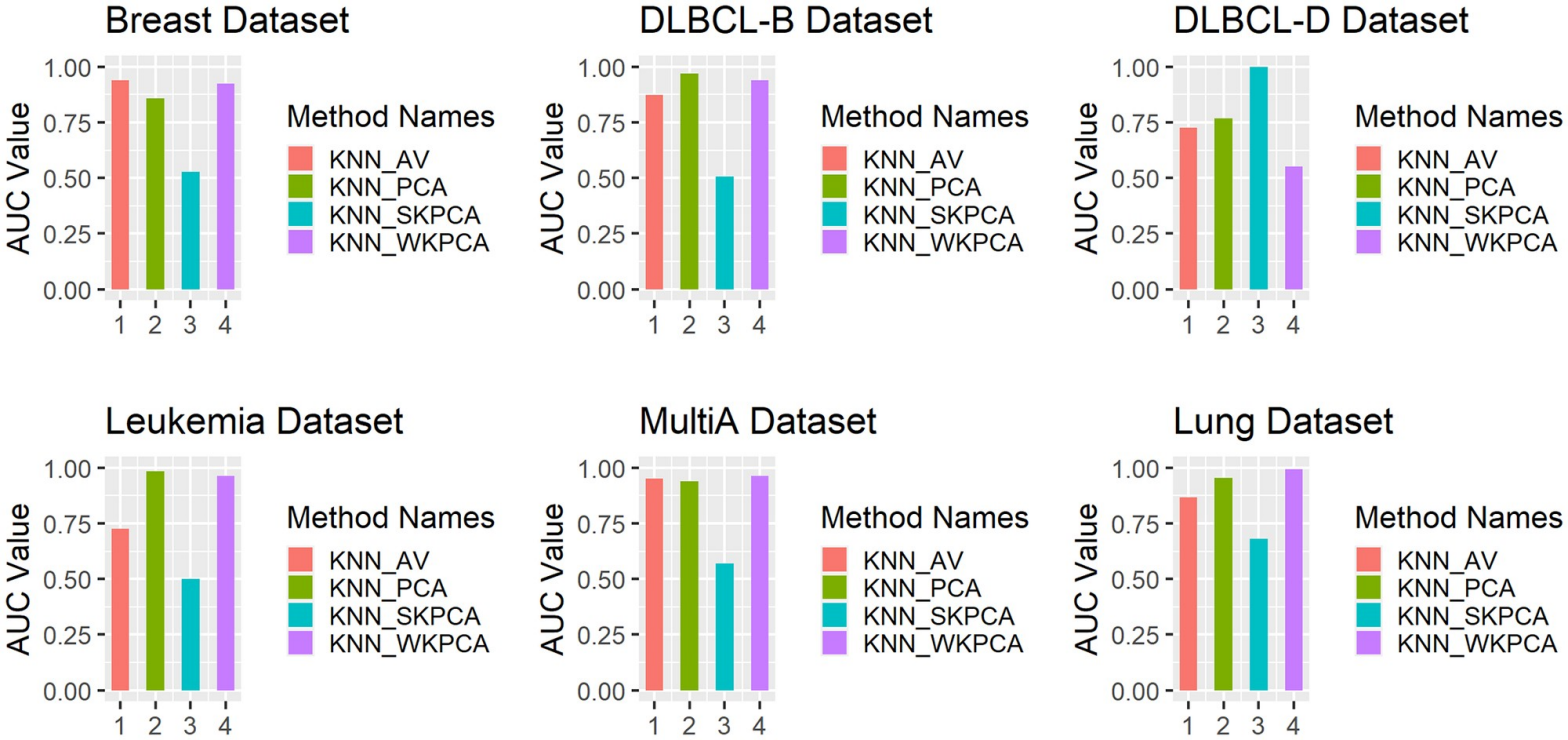
Comparison of the KNN_AV, KNN_PCA, KNN_SKPCA and KNN_WKPCA method AUC values.

To intuitively compare the classification effects of AV, PCA, SKPCA and WKPCA combined with the above five machine learning methods, the SVM, KNN and RF classifiers are taken as examples (other classifiers have similar situations). A bar chart of nested 5-fold cross-validation AUC values is drawn based on these six data sets, and the results are shown in Figs 3 to 5.

As seen from Fig 3, except that SVM_WKPCA is slightly inferior to SVM_PCA for the Leukaemia data, the AUC values of SVM_WKPCA for the other 5 data sets reach the maximum, which is a significantly better performance than those of SVM_AV and SVM_SKPCA and slightly better than that of SVM_PCA. In Fig 4, the AUC value of RF_WKPCA for the Breast data set is lower than those of RF_AV and RF_PCA, while for the other 5 data sets, the AUC values of RF_WKPCA all achieve the optimal values, but its advantage is not very significant. As seen from Fig 5, the AUC values of KNN_WKPCA reach the maximum for Multi-A and Lung data sets. The AUC value of KNN_WKPCA is similar to that of KNN_AV or KNN_PCA in Breast, DLBCL-B and Leukemia data sets. For the DLCBCL-D data set, the AUC value of KNN_WKPCA is the lowest. It is shown that for different data sets, dimension reduction using WKPCA can not make all classification algorithms achieve the optimal performance.

From Figs 3 to 5, overall it can be concluded that the AUC values of the SVM, RF and KNN classifiers can be improved after WKPCA dimension reduction for most data sets. The results show that WKPCA dimension reduction can effectively improve the predictive performance of the current mainstream machine learning classification algorithms.

## 5 Conclusion

Aiming at the characteristics of the high dimensionality, high redundancy and small sample sizes of gene expression data sets, a principal component dimension reduction algorithm based on the weighted kernel function is proposed in this paper to improve machine learning classification prediction performances and reduce the complexity of the classification process. By calculating the eigenvalues of the kernel matrix, the kernel function weight is constructed, and the t-class kernel function is also constructed to further improve the dimension reduction efficiency of WKPCA. Finally, the cumulative optimal performance rate is constructed to evaluate the overall classification level of WKPCA combined with mainstream machine learning algorithms. Through the analysis of the experimental results in 6 real data sets, compared with the all-variable model, traditional linear principal component analysis dimension reduction and single kernel principal component analysis dimension reduction, the WKPCA dimension reduction algorithm proposed in this paper can effectively improve the classification prediction performance of the current mainstream machine learning methods.

The key to WKPCA dimension reduction lies in how to choose a ‘suitable kernel function’. Our weighted kernel function makes the form of the kernel function more diversified and the selection more flexible, which allows better adaptation to data sets in different fields. In real-world problem analysis, to achieve the desired performances of machine learning on each data set in this paper, we have to attempt different kernel function combinations with different parameter settings. In other words, the best algorithm configuration is dataset-dependent. However, our WKPCA dimension reduction algorithm is quite insensitive to parameter settings.

## Supporting information

S1 File Experimental code. (ZIP)

S2 File Experimental source files. (ZIP)
